# The Effect of Monomer Size on Fusion and Coupling in Colloidal Quantum Dot Molecules

**DOI:** 10.1021/acs.nanolett.3c03903

**Published:** 2023-12-04

**Authors:** Adar Levi, Bokang Hou, Omer Alon, Yonatan Ossia, Lior Verbitsky, Sergei Remennik, Eran Rabani, Uri Banin

**Affiliations:** † Institute of Chemistry and the Center for Nanoscience and Nanotechnology, The Hebrew University of Jerusalem, Safra Campus, Givat Ram, Jerusalem 91904, Israel; ‡ The Center for Nanoscience & Nanotechnology, The Hebrew University of Jerusalem, Edmond J. Safra Campus, Jerusalem 9190401, Israel; § Department of Chemistry, University of California, Berkeley, California 94720, United States; ∥ Materials Sciences Division, Lawrence Berkeley National Laboratory, Berkeley, California 94720, United States; ⊥ The Raymond and Beverly Sackler Center of Computational Molecular and Materials Science, Tel Aviv University, Tel Aviv 69978, Israel

**Keywords:** colloidal quantum dots, quantum dot molecules, electronic coupling, size effect, atomistic pseudopotential calculations

## Abstract

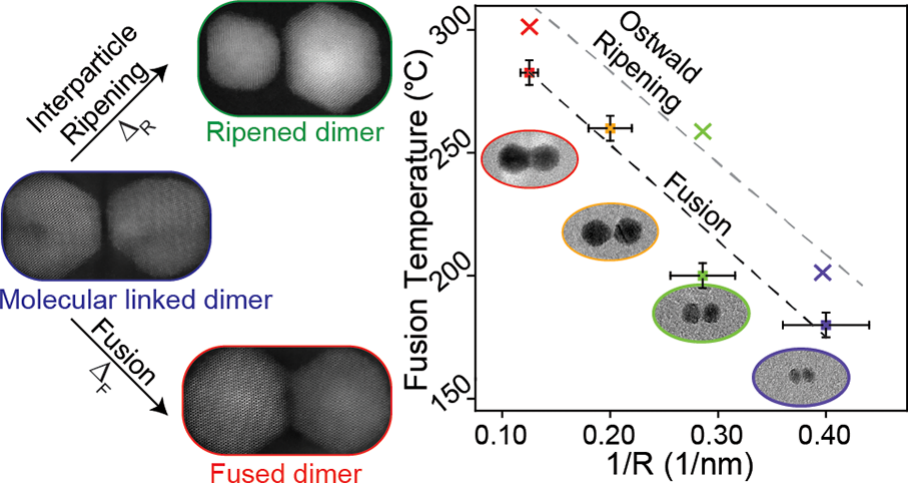

The fusion step in the formation of colloidal quantum dot molecules, constructed from two core/shell quantum dots, dictates the coupling strength and hence their properties and enriched functionalities compared to monomers. Herein, studying the monomer size effect on fusion and coupling, we observe a linear relation of the fusion temperature with the inverse nanocrystal radius. This trend, similar to that in nanocrystal melting, emphasizes the role of the surface energy. The suggested fusion mechanism involves intraparticle ripening where atoms diffuse to the reactive connecting neck region. Moreover, the effect of monomer size and neck filling on the degree of electronic coupling is studied by combined atomistic-pseudopotential calculations and optical measurements, uncovering strong coupling effects in small QD dimers, leading to significant optical changes. Understanding and controlling the fusion and hence coupling effect allows tailoring the optical properties of these nanoscale structures, with potential applications in photonic and quantum technologies.

Colloidal quantum dots (QDs), also known as artificial atoms,^1^ allow for tuning of the electronic properties through size control as dictated by quantum confinement.^2−4^ Combined by wet-chemical processing, this has led also to their widespread implementation in numerous technologies ranging from displays to biological tagging to light harvesting.^5−7^ Further promoting the artificial atom analogy, we recently introduced a solution-based approach for forming coupled quantum dot molecules (CQDMs), as a means of enhancing the properties and functionalities of such systems.^8^ Using core/shell QDs as monomers, the CQDM dimers manifest quantum mechanical coupling effects dictated by the monomer core and shell size, the relative monomer orientation, and the connecting neck characteristics. This approach provides additional flexibility in designing the system properties, more so than in other complex heterostructred systems.^9−11^ Herein, we study the effect of nanocrystal size on the fusion step of the two monomers, governing CQDM’s structural robustness and dictating the extent of electronic coupling.

Fusion in nanocrystal arrays is also of interest from the viewpoint of controlling the coupling and hence transport of charge carriers. In Pb-chalcogenide nanocrystal arrays, the softness of the lattice allowed for realizing fused QD solids via wet chemistry at room temperatures.^12^ For CdSe nanocrystal arrays, wet-chemical treatments allowed for forming connecting bridges between the constituent monomers.^13,14^ The fusion of nearby QDs also takes place in the widely studied oriented attachment process, in which two nanocrystals, often within the cluster regime, align and fuse through reactive facets, key for the formation of numerous anisotropic nanostructures.^15,16^ However, a systematic study of size effects on fusion was not yet performed, in particular, for monomers attached by an organic linker. Our study reveals distinct temperature ranges for successful fusion that vary with QD sizes, establishing a linear relationship between the fusion temperature and the inverse QD size. In large QD dimers, a dominance of [1010] facet attachment is observed, assigned to their relative abundance and relative alignment suitable for fusion. Furthermore, combining atomistic level pseudopotential calculations with optical studies, we investigate the electronic coupling and uncover a significant coupling effect resulting in a pronounced optical red shift between monomers and dimers for small QD size (5 nm). The study advances the understanding and potential applications of CQDMs.

For the study of the size effect on fusion and coupling, we focus on CdSe/CdS CQDM dimers as a model system. Wurtzite CdSe/CdS QDs covering a wide radii range from 2.5 to 8 nm were synthesized,^17^ and a template approach to form QD homodimers was utilized (Figure S1).^18^ Briefly, submicron silica spheres with thiol surface functionalization were used as templates for attaching a first layer of QDs. For all sizes aside from the smallest QDs, a thin masking silica layer was then introduced to prevent further binding.^19^ Next, the bound QDs were functionalized with tetrathiol molecules, allowing for the attachment of the added second QDs to the first QDs via the thiol linkers. The silica sphere template was subsequently removed by selective etching by HF, releasing the molecularly linked QD dimers.

To study the size dependence of the fusion behavior of the CQDM homodimers, we performed fusion reactions in solutions at controlled temperatures within the range of 180–300 °C for 20 h. A heating rate of 10 °C/min was used. This gradual approach allows for slow kinetics, and first evidence for dimer fusion is typically observed after ∼10 h. We observed by transmission electron microscopy (TEM, Figure 1a and Figure S2) fusion at temperatures dependent on the QD size, from 180 °C for 5.1 nm monomers, rising to 280 °C for 16 nm monomers. To ensure precise classification of molecular-linked dimers and fused dimers, we utilized TEM imaging at 120 kV for initial characterization and employed a ultrahigh-resolution TITAN-THEMIS with a HAADF-STEM (high-angle annular dark-field scanning transmission electron microscopy) detector at 300 kV for detailed analysis, achieving clear identification of fusion degrees. Upon further heating to higher temperatures, fragmented dimers and various sized particles were observed as interparticle Ostwald ripening dominates the reaction (ranging from 200 to 300 °C for the 5 nm to 16 nm monomers, respectively, Figure S3). Each dimer size exhibited a specific fusion temperature range in which the formation of a continuous lattice between the molecular-linked dimers is detected, resulting in the creation of a neck region. While ligand coverage influences the neck width, the overall fusion process is primarily governed by the temperature.^18,20^

**Figure 1:**
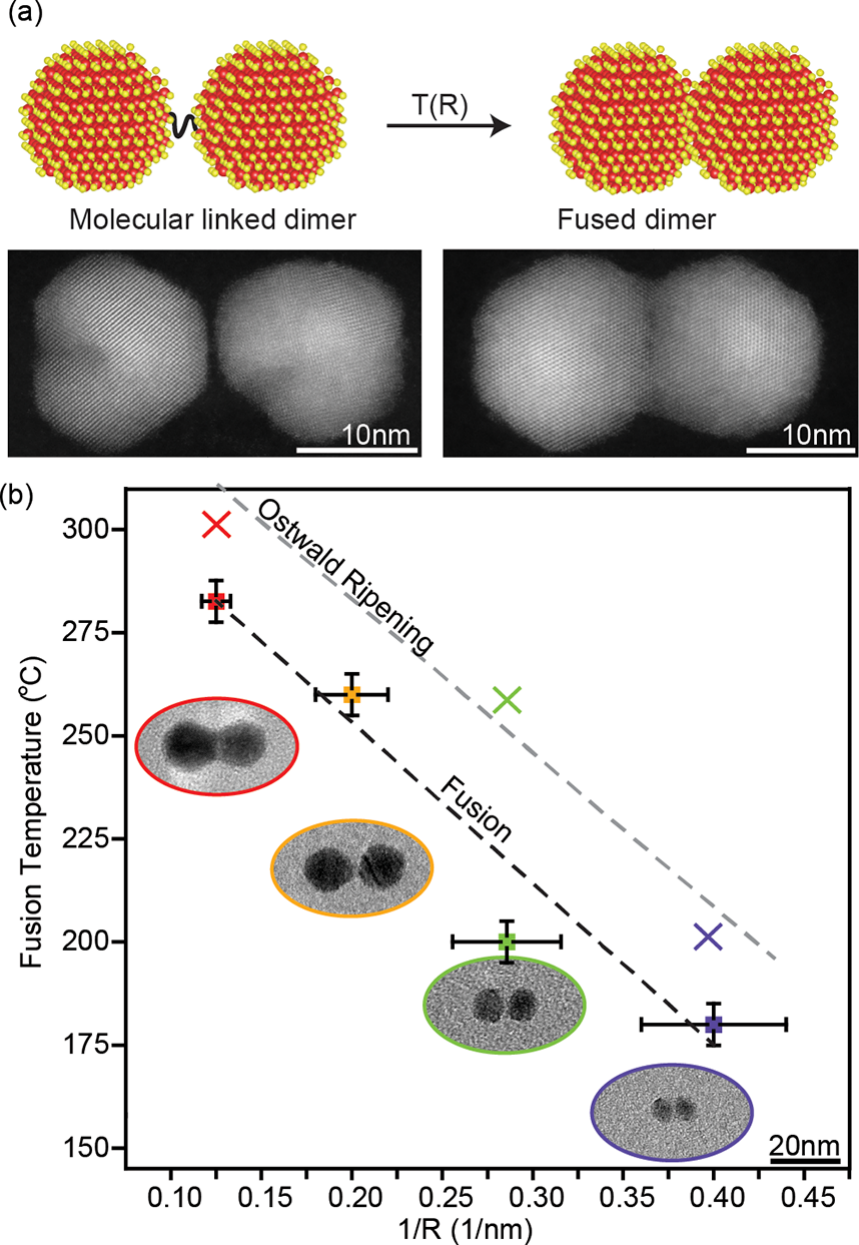
(a) The fusion process in molecularly linked dimers. The diagram illustrates the transformation from a non-fused dimer to a fused dimer using 8 nm radius QDs. HAADF-STEM images provide visual confirmation of the dimer fusion. (b) Size dependence of the fusion temperature and ripening threshold in colloidal CQDMs. The temperature is plotted against the reciprocal of the QD radius. Data points are indicated by squares (minimal fusion threshold) and crosses (maximal temperature before ripening) in different sizes: 2.5 nm (blue), 3.5 nm (green), 6 nm (yellow), and 8 nm (red). Accompanying TEM images (scaled) illustrate the structural characteristics of single dimers at each size.

Figure 1b presents the size dependence of the optimal fusion temperature (squares) as well as that of the interparticle Ostwald ripening temperature (× symbols), which refers to the temperature range at which interparticle Ostwald ripening becomes a significant factor, leading to the disintegration of dimers. Both manifest a systematic linear dependence of the related temperature on the inverse of nanocrystal radii. In all sizes, we observe a gradual transformation from non-fused dimers to fused dimers and eventually to the disintegration of dimers over a range of temperatures. The size dependence in the fusion behavior of the QDs is attributed to the interplay between surface atom diffusion and QD ripening. During fusion, the merging of the QD surfaces, via intraparticle ripening, leads to the formation of a larger interfacial area, reducing the overall surface energy of the system.^14,20,21^ However, with increasing temperature, the thermal energy activates interparticle ripening that destabilizes the fused dimer structure as well as any residual monomers. The smaller QDs dissolve and redeposit onto the larger QDs due to differences in solubility.^22^ The observed size dependence arises from the variation in the surface area to volume ratio. Smaller radius monomers and the related CQDMs exhibit a higher surface area to volume ratio compared with larger ones, resulting in a relatively higher surface energy per unit volume. Consequently, CQDMs with a smaller radius require a lower temperature (180–200 °C) before reaching the breaking point and undergoing disintegration and interparticle ripening, whereas larger radius CQDMs experience this transition only at 280–300 °C.^23^

The linear dependence of both the fusion and the ripening temperatures on the inverse of the nanocrystal radii is consistent with the interplay of fusion and ripening. A similar trend was also observed in the size-dependent melting-point behavior in metal and semiconductor nanocrystals.^23^ The intercept corresponding to the bulk regime is at ∼320 °C for the fusion temperature, in agreement with the solution annealing temperature during the synthesis of the native QDs.^17^ At this temperature, defects and lattice inhomogeneities are removed by atom diffusion from the CdSe/CdS QDs, resulting in the formation of a stable crystal structure.^24^ This implies a relation between the fusion process of QD homodimers and the annealing process of monomers, where atoms in the lattice diffuse and rearrange, supporting the major influence of surface energy and ripening mechanisms in the dimer fusion and breaking processes, respectively.

An additional interesting observation is obtained from the structural analysis of the largest CQDMs constructed from the giant CdSe/CdS core/shell QDs (overall diameter of 16 nm), fused at 280–300 °C. We utilized both HRTEM as well as HAADF-STEM to obtain atomic-resolution images of the icosahedral-shaped QDs (Figure S4 shows HRTEM analysis for monomers, as illustrated in Figure 2d, inset).^25^Figure 2a shows the HAADF-STEM image of a dimer, alongside the FFT of a selected area of either monomer, revealing that both are viewed down the [0001] *c*-axis. Therefore, this is a case of homonymous fusion via the [1010] facets illustrated by the atomistic model. Other cases were also observed, albeit much less abundant: e.g., a dimer with homonymous attachment via the [1011] facet in which the fusion area appears to be small and limited by the facet size and by the tilted orientation (Figure 2b). Interestingly, cases of heteronymous attachment (Figure 2c) were rare (8 out of 58 dimers). Overall, the dominance of homonymous attachment through the [1010] facet was observed (Figure 2d). This differs from prior results for CQDMs constructed from smaller monomers, 7 nm in diameter, where heteronymous versus homonymous attachments were equally abundant.^18^ The fused dimers manifested dominant fusion through either the reactive [0002] facet or [1010] facet which is less reactive yet sterically accessible. This difference in fusion behavior further highlights the size effect on this process and its mechanism.

**Figure 2:**
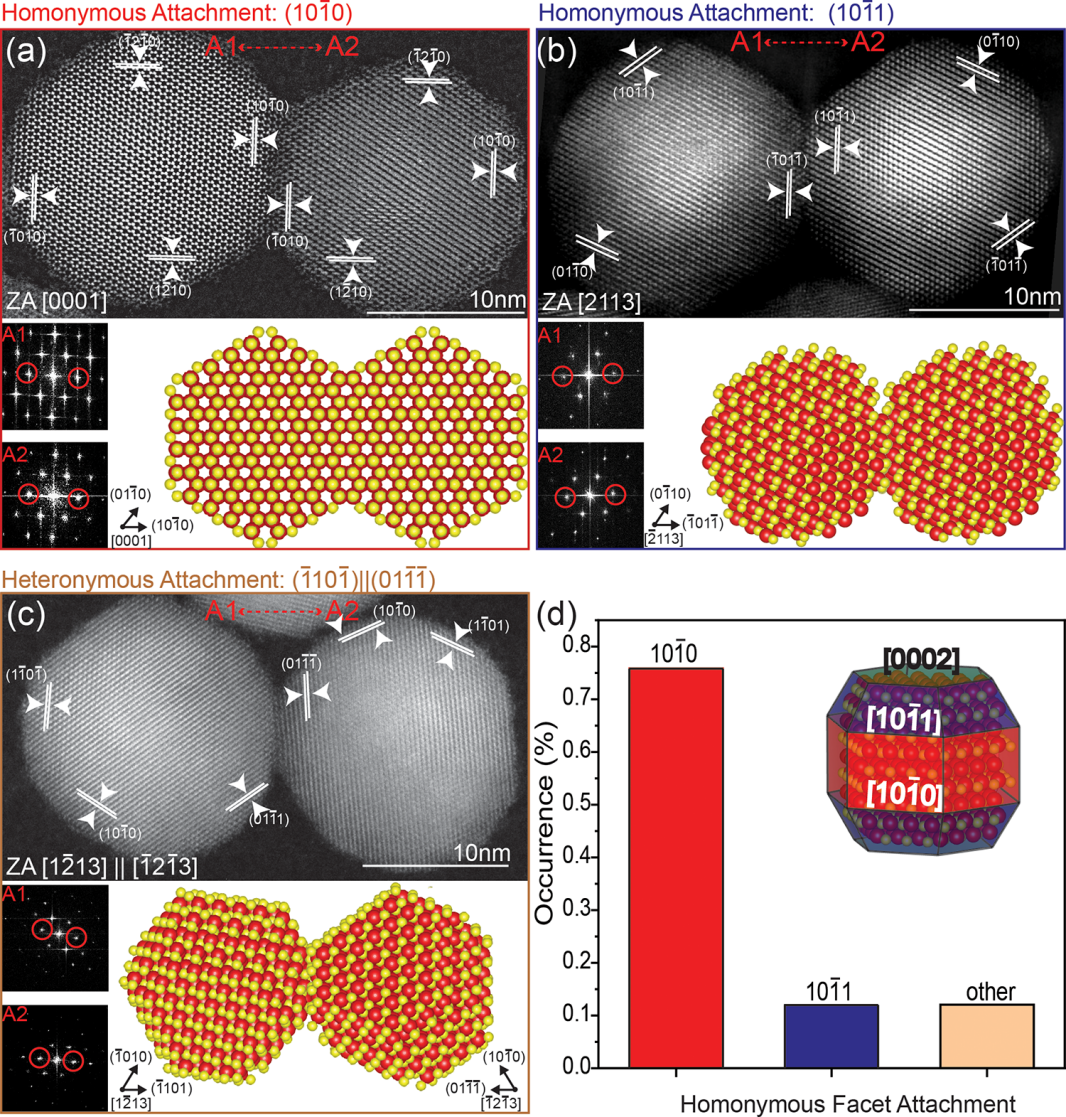
Fusion orientation relationships in the 8 nm radius CdSe/CdS core/shell CQDMs. HAADF-STEM images showing the homoplane attachment, along with their corresponding FFT patterns and atomic models. The observed facet orientation relationships are depicted as (a) (1010)||(1010), (b) (1011)||(1011). (c) The respective heteroplane attachment, (1101)||(0111). (d) The statistical distribution of the observed facets involved in both homonymous attachments. The inset illustrates the QD’s atomic model and its dominant facets.^28^

The attachment preference through the side facet for giant CQDMs is attributed to two consecutive steps in the dimer formation process. First, during the connection of two QDs using the tetrathiol linker over the silica template, the dense coverage of the linker on one side of each QD is more likely to occur on the [1010] facet due to its larger surface area compared to other facets. Next, during the fusion process at elevated temperatures, there is no preference for polar attachment through the [0002] facets, as is typically observed in wurtzite structure anisotropic growth and oriented attachment.^26^ Moreover, the nonpolar nature of the [1010] facet reduces the requirement for a head-to-tail arrangement of the QDs, which is less probable.^27^ Therefore, the combination of the nonpolar nature and larger surface area of the [1010] side facet promotes its selectivity and dominance in the fusion process. This may also contribute to the low abundance of heteronymous faceted dimers, since there is a higher chance that a [1010] facet will be linked to an identical facet rather than another facet. Another important factor is the high fusion reaction temperature in the giant CQDMs versus the smaller ones (280 °C vs 180 °C, respectively). At this higher temperature, weakly linked dimers, with incompatible facets, will break due to their instability. Only the robust well-aligned dimers remain stable and fuse. In this dominant case of attachment through the [1010] facets, we also find that the connecting neck width is ∼8–9 nm, indicating complete facet fusion (Figure S5). Such dominant homonymous attached CQDMs connected via the [1010] facet provides uniform bridging between the QDs, which in turn dictates the degree of coupling.^20^

By identifying the appropriate fusion temperature for each size category, we achieved precise control over the core–core distances in the resulting homodimers. This control was crucial in examining the coupling effects within CQDMs,^29,30^ where stronger coupling manifested by a larger optical redshift is expected for smaller core–core distances. First, we conducted energy-dispersive X-ray spectroscopy (EDS) analysis on CdSe/CdS CQDMs of various sizes. The EDS mapping shows that selenium predominantly occupied the QDs’ core, while sulfur was primarily present in the shell and cadmium was distributed in both the core and shell. This elemental distribution remained consistent regardless of the dimensions of the CQDMs and the corresponding fusion temperature (Figure 3a,b), showing that the core/shell architecture was maintained. Moreover, an EDS line scan along the dimer axis illustrates the dimer architecture and provides information on the core–core distance based on the selenium signal (Figure 3c,d). Reducing the QD size led to a decrease in the core–core distance within the CQDMs, and when the core is symmetrically located inside the shell, the core–core distance is equal to the diameter of the QDs. For example, in the case of 2.2/5.8 nm (core/shell) CQDMs, the distance between the cores was approximately 16 nm, while for 1.4/2.1 nm CQDMs, the distance decreased to 7 nm.

**Figure 3:**
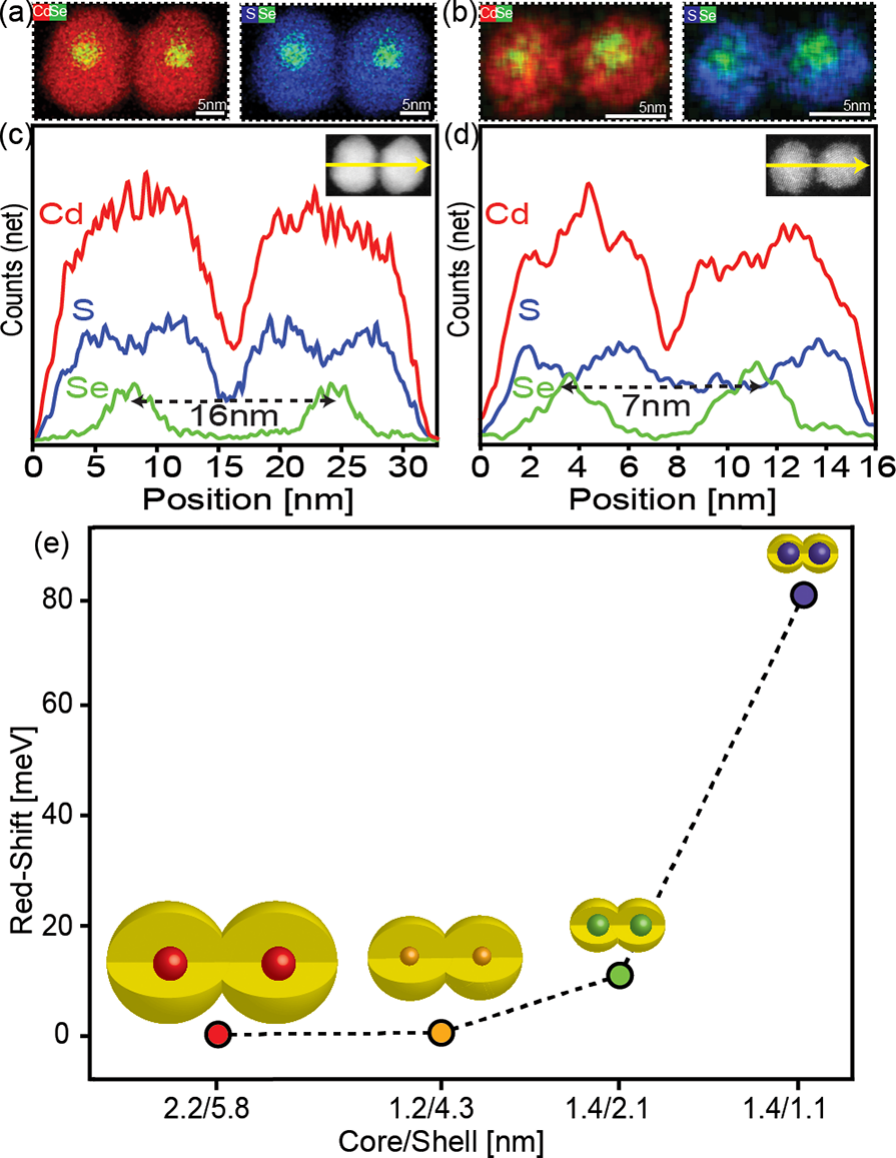
EDS analysis and coupling strength in CQDMs of different dimensions. (a, b) EDS mapping of CdSe and SSe, in 2.2/5.8 and 1.4/2.1 nm CQDMs, respectively. (c, d) EDS line scans showing elemental composition and core–core distance in the CQDMs. The coupling strength (e), measured by a redshift in optical spectra, increases with decreasing core–core distance. Results highlight the influential role of core–core distance in determining the coupling strength in CQDMs.

The electronic coupling effects in the different-sized dimers were studied experimentally by examining the optical absorption and emission spectra in comparison to the respective monomers (Figure 4 and Figure S6, for the smallest and larger CQDMs, respectively). Comparing monomers and dimers, the absorption near the band edge region is typically broadened and smeared in the latter, while the oscillator strength (per particle) at short wavelengths is essentially doubled, as expected. The extent of the redshift, observed in the absorption onset but best resolved from comparing the monomer versus dimer PL, is a relevant measure of coupling (Figure 3e). The two larger CQDMs with a greater core–core distance display a negligible redshift between monomer and dimer PL. It is important to note that, in addition to the core–core distance, the core/shell band alignment of the different-sized CQDMs may also influence the coupling strength. CQDMs with a large core and thick shell (e.g., 2.2/5.8 nm) show less coupling due to the significant distance between the interacting cores and the localization of the electron wavefunction in the core. Similarly, CQDMs with a small core and thick shell (e.g., 1.2/4.3 nm) exhibit negligible coupling despite their quasi type II band alignment,^31^ with the core–core distance playing a dominant role. The smaller CQDMs with dimensions of 1.4/2.1 and 1.4/1.1 nm, characterized by quasi type II band alignment, display monomer to dimer redshifts of 16 and 86 meV, respectively. These examples highlight the prominent role of core–core distance in determining coupling strength, along with other factors such as band alignment and confinement effects that are highly sensitive to the QD size.^30^

**Figure 4:**
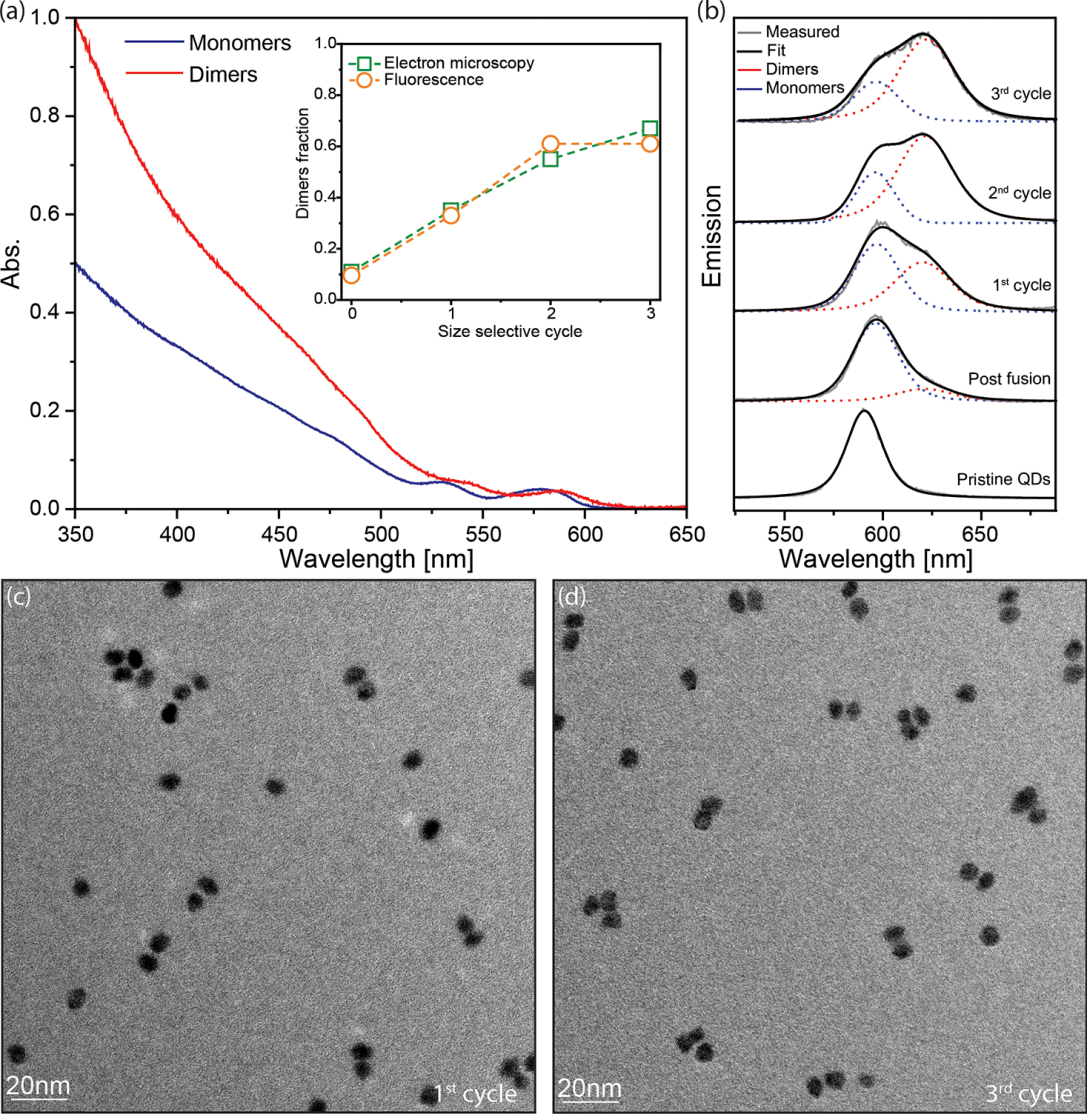
Optical characterization of CQDMs. (a) Absorption spectra of dimers (red) and monomers (blue), showcasing the notable redshift and higher absorption at higher energies in the dimers. The inset shows the fraction of dimers versus size-selective cycles based on TEM (green) and emission (orange) analysis. To extract this latter value, PL spectra were fit by two peaks (Voigt function) with fixed central wavelength and full width at half-maximum. The dimer fraction is extracted from the relative weight of its PL peak, compared with the entire emission, while dividing its contribution by 2 in light of its doubled absorption coefficient compared to monomers at the excitation wavelength. (b) Emission spectra illustrating the evolution of the emission characteristics, fitted to the two Voigt functions (gray) and differentiation between monomers (red) and dimers (dimers) with each size-selective cycle. The spectrum transitions from pristine QDs to a mixture of QDs and CQDMs after fusion, followed by size-selective separation. (c, d) TEM images showcasing the enhanced yield of dimers from the first to the last cycle of the size-selective process.

We focus on the smallest, 1.4/1.1 nm CQDMs manifesting a large redshift indicative of a high degree of coupling. Chemically, they were the least stable and required careful tuning of the fusion to achieve dimers without ripening. They manifest clear shifts in the ensemble level, allowing us to directly showcase the purification process. Notably, in the ensemble absorption spectra, we discerned a significant redshift of 36 meV (from 577 to 587 nm) at the band edge of the purfied dimer sample, relative to that of the monomers, already serving as an indication of the coupling. Furthermore, the absorption optical density at higher energies is doubled for the fused sample, as expected for dimers (Figure 4a).

Shifting our attention to the PL (Figure 4b), post fusion prior to size-selective purification, a dominant peak, red-shifted from the pristine monomers by 17 meV, is seen. This shift is assigned to slight growth of the monomers, by ∼0.2 nm, far less than one monolayer, due to the presence of excess Cd precursors, consistent with the TEM analysis for monomer size post fusion, which does not show a clear change. Notably, the shape of the post fusion PL spectrum is modified, and a shoulder, red-shifted by 86 meV from the main peak, is discerned. By employing size-selective purification cycles, the dimer to monomer fraction is increased (TEM in Figure 4c,d for cycles 1 and 3, respectively). This is resolved also by the change in the PL spectra, where a clear red-shifted peak is observed after the first purification cycle, and its relative contibution increased after the second purification cycle. This enables, on the ensemble level, clear separate measurement of the emission characteristics of the dimers and monomers.

The fraction of dimers out of the total number of particles in the samples from the TEM analysis is in excellent agreement with the dimer fraction extracted from its relative contribution to the PL spectrum (Figure 4a, inset), further supporting the assignment of the red-shifted peak to dimer emission, indicative of the strong coupling. Note that as we avoided the silica masking step in this dimer synthesis due to the small monomer size, the dimer fraction prior to purification is relatively small. Masking is performed in the dimer synthesis in larger sizes, thus blocking monomer binding on the silica surface during the second monomer addition and resulting in a much larger dimer fraction initially.^18,20^

For quantitative insight into the optical redshift in the 5 nm CQDMs, we used atomistic pseudopotential calculations^32,33^ combined with the Bethe–Salpeter equation^34^ (BSE) which includes the electron–hole interaction (details in the Supporting Information). This allowed us to delineate the shape dependence of the optical gap redshift on neck width, core-to-core distance, core radius, and shell thickness (Figure 5). The 5 nm CQDMs are less faceted than the larger CQDMs, which makes it hard to determine the attachment plane. Thus, we consider attachments for both [1010] and [0001] facet orientations. The optical redshift (δ*E*_*x*_) is defined as the difference between the first excitonic transition of the QD monomer and the corresponding CQDMs. The attachment via the [0001] direction generally has a larger redshift than attachment through the [1010] facet (Figure 5a and Figure S7, respectively). This is mainly due to the difference in hybridization energy (Figure S8), resulting from larger electronic wavefunction overlaps in the [0001] facet (Figure S9). In both attachments, the extent of neck filling has a significant effect on the redshift.

**Figure 5:**
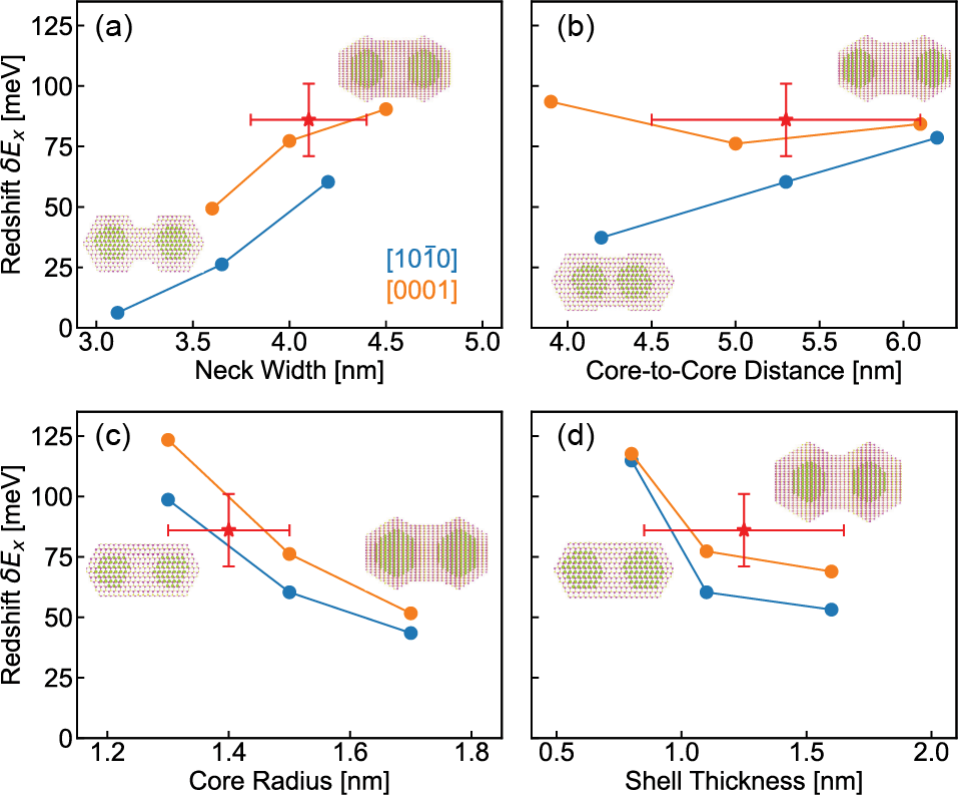
Redshift of the optical gap in 5 nm CQDMs as a function of (a) neck width, (b) core-to-core distance, (c) core radius, and (d) shell thickness. Only one parameter is changed at a time. Attachments through [1010] and [0001] facets are represented in blue and orange, respectively. Experimental data is indicated by a red star with error bars.

As depicted in Figure 5b, the redshift has a weaker dependence on the core-to-core distance in the [0001] facet and even increases for [1010] attachment while maintaining the total dimer dimensions (Figure S10). This can be explained by the increase of deconfinement energies as the two cores are separated apart, consistent with our previous study.^30^Figure 5c (Figure 5d) presents the dependence on core radius (shell thickness) while keeping the shell thickness (core radius) fixed for the case of a “full neck” (Figures S11 and S12). δ*E*_*x*_ decreases for a larger core radius or shell thickness, showing good agreement with the trends observed in Figure 3e, while the calculations focus on the strong coupled regime attained for the small size core/shell QDs. In all panels of Figure 5, we found good agreement between the experimentally observed dimer red shift (red symbol) and the calculations. Particularly, the CQDM in the [0001] facet attachment is in agreement with the calculations within experimental error. For dimers attached through [1010], good agreement with the measured optical shift is observed as well when the core radius is somewhat smaller. Considering the inherent error in the sizing of the core/shell monomers, the calculations thus provide valuable insight into the sensitivity of coupling to the various structural parameters.

In summary, the fusion temperature for CQDMs increased linearly with the reciprocal QD radius. This size dependence, seen also in nanocrystal melting and in ripening of monomers, which takes place at a higher temperature, indicates the importance of the surface energy for fusion. The proposed fusion mechanism involves intraparticle ripening where atoms migrate toward the reactive neck region. Interestingly, a pronounced preference for attachment through the [1010] facet in the large dimers is observed, unlike smaller ones for which a broad distribution for the fusion orientation prevails. This relates to the higher fusion temperature for the large CQDMs, under which just the robust organic linked dimers remain intact, alongside the relatively high abundance of those facets. Finally, CQDMs of small monomers manifest a pronounced red shift in the ensemble optical spectra compared to monomers, and the extent of the shift is consistent with atomistic calculations indicating strong coupling. This understanding of the fusion behavior and its size dependence also bears relevance to CQDMs constructed of different materials as well as for other types of assemblies such as QD superlattices.

## Supporting Information Available

Experimental details, methods, theoretical modeling, and additional figures and a table (PDF)
