# Supplementary material for: The Effect of Monomer Size on Fusion and Coupling in Colloidal Quantum Dot Molecules
Source: Nano Lett. 2023 Dec 4;23(23):11307–13. doi: 10.1021/acs.nanolett.3c03903 (PMC11145643; doi:10.1021/acs.nanolett.3c03903)
Supplement: Supplementary file 1 — nl3c03903_si_001.pdf [file nl3c03903_si_001.pdf]

Supplementary Information for

The Effect of Monomer Size on Fusion and Coupling in Colloidal Quantum Dot Molecules

Adar Levi, Bokang Hou, Omer Alon, Yonatan Ossia, Lior Verbitsky, Sergei Remennik, Eran Rabani*, Uri Banin*

Corresponding authors: eran.rabani@berkeley.edu & uri.banin@mail.huji.ac.il

CONTENTS

Experimental Section 2

Theoretical Modeling 4

Figure S1: 6

Figure S2: 7

Figure S3: 8

Figure S4: 9

Figure S5: 10

Table S1: 11

Figure S7: 12

Figure S8: 13

Figure S9: 14

Figure S10: 15

Figure S11: 16

Figure S12: 17

EXPERIMENTAL SECTION

Chemicals

Oleylamine (OAm, 70%), oleic acid (OA, 99%), 1-octadecene (ODE, 90%), cadmium oxide (CdO, ⩾99.99%), selenium (Se, 99.99%), trioctylphosphine oxide (TOPO, 99%), 1-octanethiol (⩾98.5%), pentaerythritol-tetrakis(3-mercapto-propionate) (PTMP 95%), ammonia aqueous (28.5%), tetrahydrofuran (THF, ⩾99.9%), N-methylformamide (NMF, 99%), toluene (99.8%), hydrofluoric acid (HF, 48%), polyvinylpyrrolidone (PVP, 10k), ethanol (99%), tetraethyl orthosilicate (TEOS, 98%), hexane (95%) and (3-Mercaptopropyl) trimethoxysilane (MPTMS, 95%) were obtained from Sigma Aldrich. Trioctylphosphine (TOP, 97%) was purchased from Strem Chemicals. Octadecylphosphonic acid (ODPA, >99%) was purchased from PCI synthesis. All the reagents were used as received without further purification.

Quantum dots synthesis

The CdSe core growth: process involved the addition of 60 mg CdO, 280 mg ODPA, and 3 g TOPO to a 50 mL flask. The mixture was then heated to 150°C and degassed under vacuum for 1 hour. Subsequently, under argon flow, the reaction mixture was further heated to 320°C, resulting in the formation of a colorless clear solution. Following the addition of 1.0 mL TOP to the solution, the temperature was raised to 350°C. At this stage, a swift injection of a Se/TOP solution (60 mg Se in 0.5 mL TOP) took place. The reaction was allowed to proceed at 350 °C for a suitable period and was then stopped by removing the heating mantle. Finally, the resulting CdSe particles were precipitated using acetone and dispersed in 3 mL hexane, creating a stock solution for further use.

CdS Shell growth: to initiate the shell growth, a hexane solution containing 200 nmol of CdSe core was added to 50 mL flask containing 6 mL ODE, and 6 mL OAm. The solution was then degassed under vacuum at room temperature for 30 minutes, followed by an additional 30 minutes at 90 °C to remove any residual hexane, water, and oxygen present. Subsequently, the reaction solution was heated to 310 °C under an argon flow with continuous magnetic stirring. During the heating process, when the temperature reached 240 °C, a predetermined amount of cadmium (II) oleate (Cd-oleate, diluted in 6 mL ODE) and octanethiol (1.2 equivalent amounts to Cd-oleate, diluted in 6 mL ODE) were dropwise injected into the growth solution at a rate of 3 mL/h using a syringe pump. Following the infusion of the precursors, 2 mL of oleic acid was quickly injected, and the solution was further annealed at 310 °C for

30 minutes. The resulting CdSe/CdS core/shell CQDs were precipitated by adding ethanol, and then dispersed in hexane. To ensure further purification, two additional precipitation-dispersion cycles were performed. Finally, the particles were suspended in approximately 2 mL of hexane for subsequent use.

Coupled quantum dots formation

SiO2-SH NPs synthesis2: The SH-SiO2 NPs were synthesized in a two-step process. Initially, a flask containing 180 mL of ethanol and 17 mL of NH3•H2O (28%) went through stirring at a rate of 1200 rpm/min for 5 minutes. Subsequently, 5 mL of TEOS was introduced into the reaction solution. The mixture was stirred for 10 hours, followed by the injection of 1.5 mL of MPTMS. The stirring was continued for an additional 10 hours. Finally, the SH-SiO2 NPs were collected through centrifugation and dispersed in 31 mL of ethanol.

Formation of SiO2@CdSe/CdS dimers: The CdSe/CdS QDs dimer structure was synthesized following our previous method and is based on a generic approach. Initially, 1 mL of SH-SiO2 NPs was combined with 0.1 mL of a PVP/ethanol solution (20 mg/mL) and allowed to mix for 1 hour. The resulting mixture was then dispersed in 3 mL of toluene. Simultaneously, 2 nmol of wurtzite CdSe/CdS QDs were mixed with the purified SiO2-PVP solution and left to react for 1 hour, yielding the CdSe/CdS@SiO2 composite. After undergoing two wash cycles, the CdSe/CdS@SiO2 composite was dispersed in 5 mL of ethanol. Subsequently, 330 μL of ammonia solution (28.5% wt %) was added to the solution and stirred for 5 minutes. Following this, 20 μL of TEOS was slowly injected using a syringe pump over a period of 2 hours. After an additional 3 hours of stirring, the samples were collected through centrifugation and dispersed in 5 mL of THF, resulting in the formation of SiO2@CdSe/CdS@SiO2 particles with a surface layer of silica for masking and immobilization the QDs. Next, 100 μL of PTMP was added to the solution and stirred for 10 hours. After two rounds of centrifugation, the samples were dispersed in 5 mL of THF. Subsequently, 2 nmol of CdSe/CdS QDs were dropwise added and stirred for 3 hours. Finally, the obtained dimer CdSe/CdS@SiO2 structures were wash two times using centrifugation.

Etching and releasing of CdSe/CdS: Briefly, the dimer CdSe/CdS@SiO2 was combined with 2 mL of HF/NMF (15%) solvent and stirred in an oil bath at 60 °C for 10 hours. Subsequently, the CdSe/CdS QDs dimers were collected through centrifugation and dispersed in 2 mL of ethanol.

Fusion of CdSe/CdS dimers: CdSe/CdS dimer (in 2 mL of ethanol) were mixed with 3 mL of ODE, 300 μL of Cd-oleate (0.2 M), and 50 μL of OAm and 10 μL of OA. The reaction solution was degassed under vacuum at room temperature for 10 min and again at 120 °C for an additional 30 min. Later, the reaction mixture was heated to the desired fusion temperature based on the QDs size for 20 h under argon flow. The resulting fused particles were precipitated with ethanol and dispersed in 2 mL toluene.

Characterization

Absorption spectra were measured using a Jasco V-570 UV-Vis-NIR spectrophotometer. Fluorescence spectra was measured with a fluorescence spectrophotometer (Edinburgh instruments, FL920). Transmission electron microscopy (TEM) was performed using a Tecnai G2 Spirit Twin T12 microscope (Thermo Fisher Scientific) operated at 120 kV. High-resolution TEM (HRTEM) measurements were done using Tecnai F20 G2 microscope (Thermo Fisher Scientific) with an accelerating voltage of 200 kV. High-resolution scanning-transmission electron microscopy (STEM) imaging and elemental mapping was done with Themis Z aberration-corrected STEM (Thermo Fisher Scientific) operated at 300 kV and equipped with high angular annular dark field detector (HAADF) for STEM and Super-X energy dispersive X-Ray spectroscopy (EDS) detector for high collection efficiency elemental analysis. The images and EDS maps were obtained and analyzed with Velox software (Thermo Fisher Scientific).

THEORETICAL MODELING

The QD monomers were constructed by adding CdS monolayers to a CdSe core cleaved from bulk crystal with a lattice constant wurtzite CdSe (*a*=4.3 Å, c=7.0 Å). The CdSe/CdS QDs dimer were built by attaching two monomers either through the $[10\bar{1}0]$ or $[0001]$ facet. The dimension of neck between two monomers can be controlled by adding/removing CdS monolayers in the connection area. The structures were optimized with Stillinger-Weber force field for II-VI nanostructures using the conjugate gradient minimization implemented in LAMMPS. The structures of Figure 5 are shown in Figure S7-10.

The semi-empirical pseudopotential model was used to describe the quasiparticle Hamiltonian and the filter diagonalization technique was applied to calculate the eigenstates of the dimer near the bandgap. The screened strain-dependent pseudopotentials were fitted to the functional form in the momentum space

$$\tilde{v}(q) = a_0[1 + a_4\mathrm{Tr}\epsilon]\frac{q^2 - a_1}{a_2\exp(a_3 q^2) - 1}$$

where q is the momentum, ϵ is the strain tensor, and the pseudopotential parameters for Cd, Se, and S are collected in Table S1. All parameters were fitted to reproduce bulk CdSe and CdS band structures, band gaps, and effective masses. The real-space quasi-particle Hamiltonian is given by

$$h_{\mathrm{QP}}(\boldsymbol{r}) = -\frac{1}{2}\nabla_r^2 + \sum_{\mu} v_{\mu}(|\boldsymbol{r} - \boldsymbol{R}_{\mathbf{0},\mu}|),$$

where ν_{μ} is the real-space pseudopotential for each atom type. The filter-diagonalization technique was then applied to obtain N_e quasiparticle electron states $\phi_a(\mathbf{r_e})$ and N_h hole states $\phi_i(\mathbf{r}_h)$ near the band gap. The calculations were implemented on real-space grids less than 0.8 a.u. such that the eigenenergies converges less than 10^{-3} meV.

The exitonic states $\psi_n(r_e, r_h)$ are given by combining the quasiparicle states

$$\psi_n(r_e, r_h) = \sum_{a}^{N_e}\sum_{i}^{N_h} c_{a,i}\,\phi_a(\mathbf{r}_e)\phi_i(\mathbf{r}_h),$$

where the coefficients are determined by solving the Bethe-Salpeter equation with static screening approximation (dielectric constant ε=6.0). Figure S11 shows the projected density of $|\psi_n(r_e, r_h)|^2$ in CQDM with different neck sizes. The lowest excitonic energy is defined as E_x, and the excitonic energy shift between the QD dimer and monomer is $\delta E_x = E_x^{\mathrm{monomer}} - E_x^{\mathrm{dimer}}$. The fundamental gap hybridization $\Delta_g = \frac{1}{2}(E_{e2} - E_{e1} + E_{h1} - E_{h2})$, with E_{e1}, E_{h1} being the LUMO and HOMO energies respectively (see Figure S12). This characterizes the hybridization energies of noninteracting electrons and holes near the band edge.

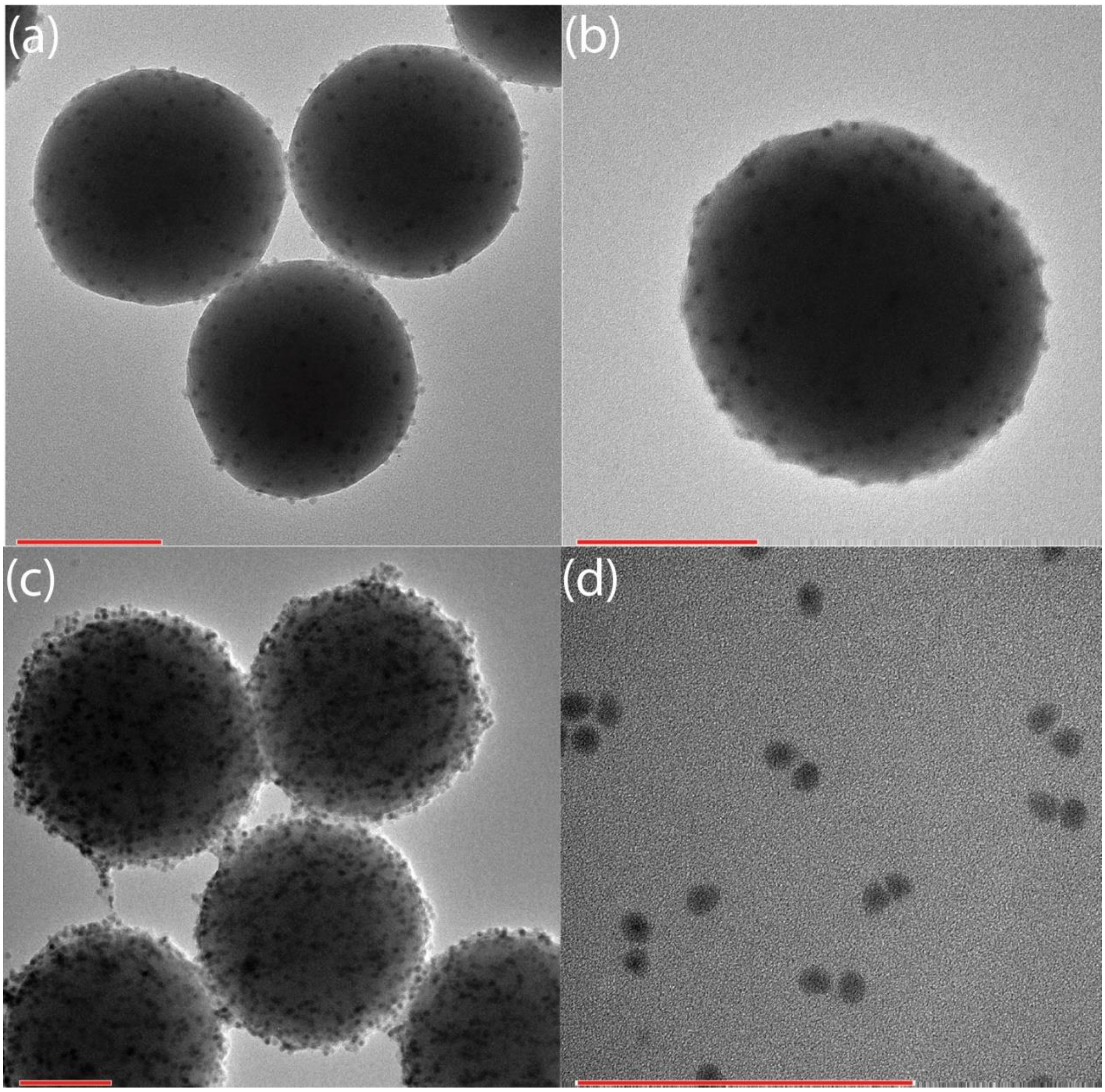

FIGURE S1: Chart of the coupled CQDs molecule fabrication steps: illustrates the consecutive steps involved in the formation of dimers using the silica template approach. The process begins with the binding of the QDs to the silica template, as shown in (a). Subsequently, the binding sites on the silica surface are masked, as depicted in (b). This step is followed by a ligand exchange process, where a molecular linker is used to facilitate the attachment of a second layer of QDs onto the SiO2@CdSe/CdS structure, as shown in (c). Finally, the silica template is etched using an HF solution, resulting in the formation of non-fused dimers. The scale bar in the figure represents 100 nm.

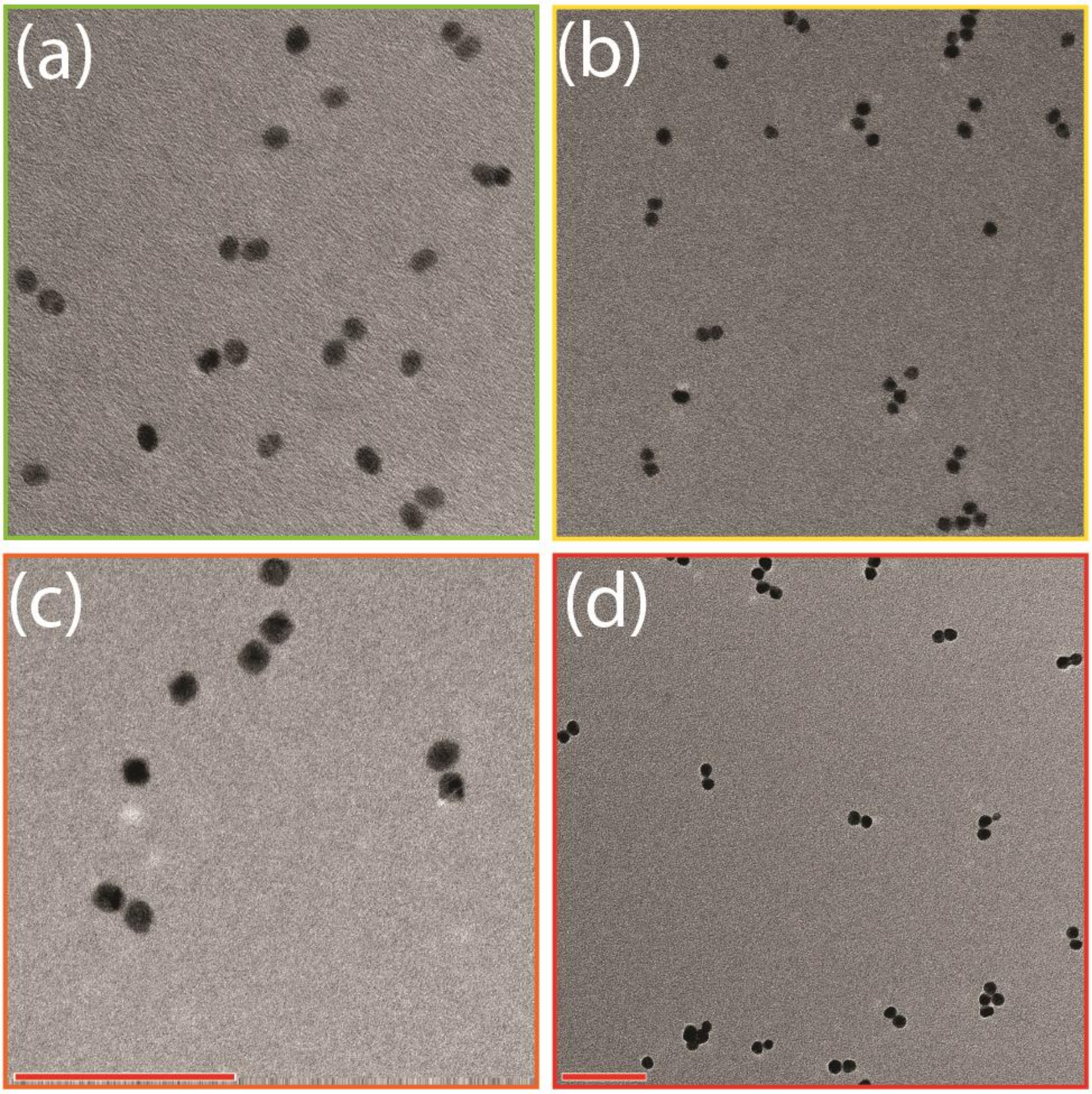

FIGURE S2: TEM micrograph of CdSe/CdS fused QDs with varying sizes: The TEM micrograph showcases CdSe/CdS CQD molecules with different sizes achieved through a fusion process by increasing the synthesis temperature for the larger quantum dots. The specific sizes for each CdSe/CdS core/shell are as follows: (a) 1.4/1.1 nm (b) 1.4/2.1 nm (c) 1.2/4.3 nm and (d) 2.2/5.8 nm. The scale bar in the figure represents 100 nm.

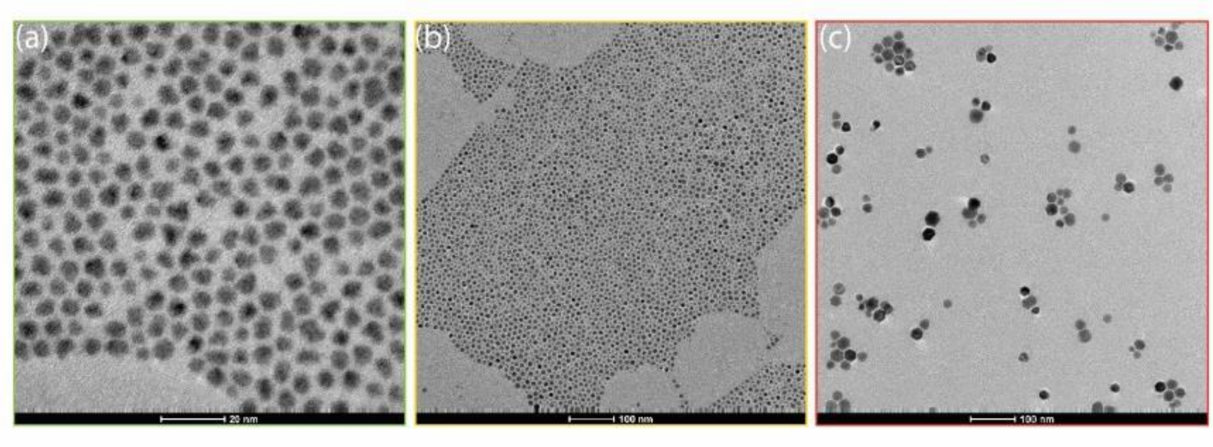

FIGURE S3: Inter-particle ripening of the CQD dimer molecules. TEM images for the fused dimer with inter-particle ripening when the temperature raised above the critical fusion temperature. The specific sizes for each CdSe/CdS core/shell are as follows: (a) 1.4/1.1 nm at 200°C (b) 1.4/2.1 nm at 260°C and, (c) 2.2/5.8 nm at 300°C

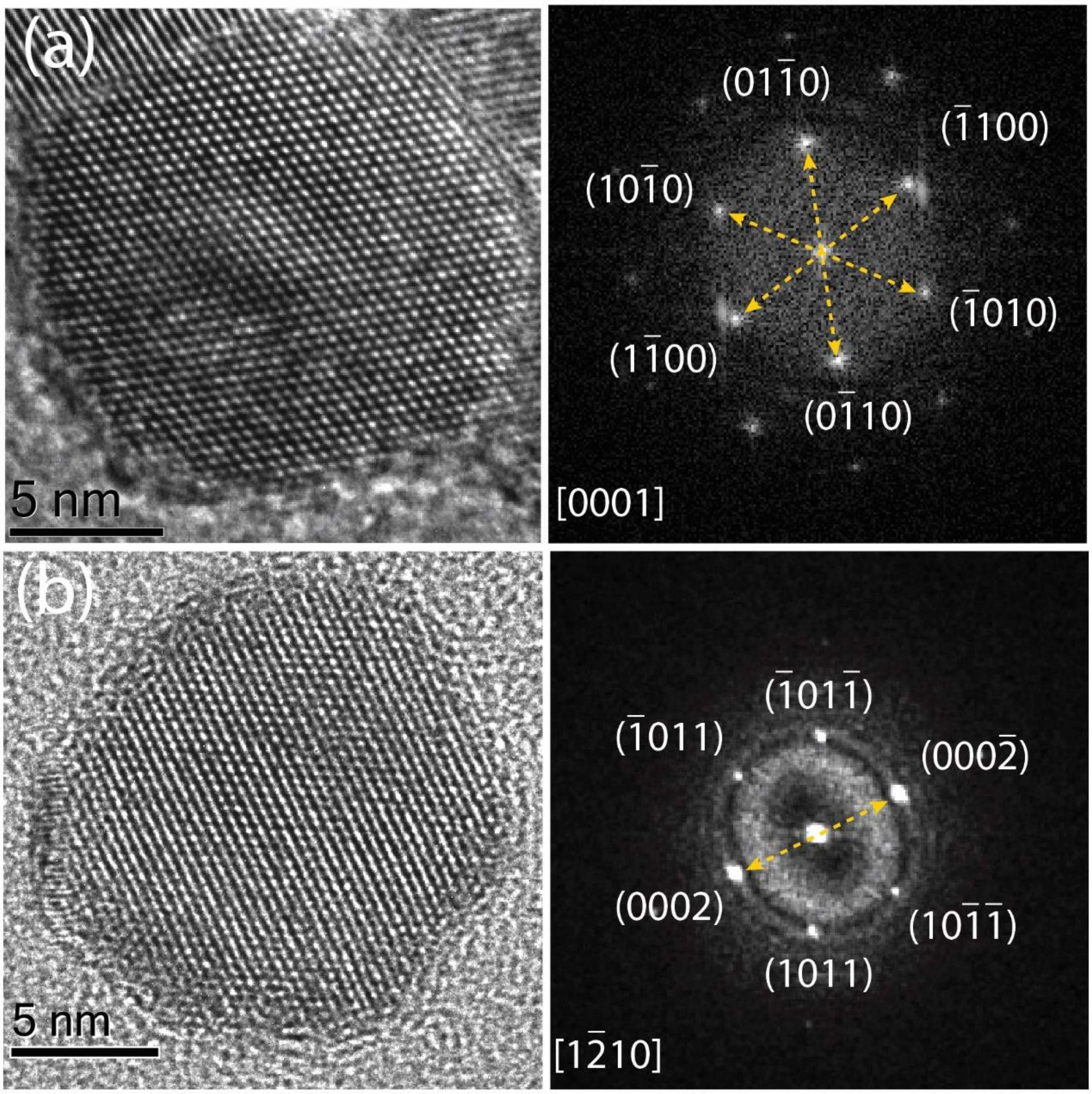

FIGURE S4: Structural Characterization of the CQDs. HR-TEM micrograph and its Fourier transform pattern analysis of wurtzite CdSe/CdS core/shell QDs with a size of 2.2/5.8 nm, viewed under different zone axes: (a) [0001], showing the dominance of the side facets$\{10\bar{1}0\}$, and (b) $[1\bar{2}10]$, depicting the c-axis direction plane (0002).

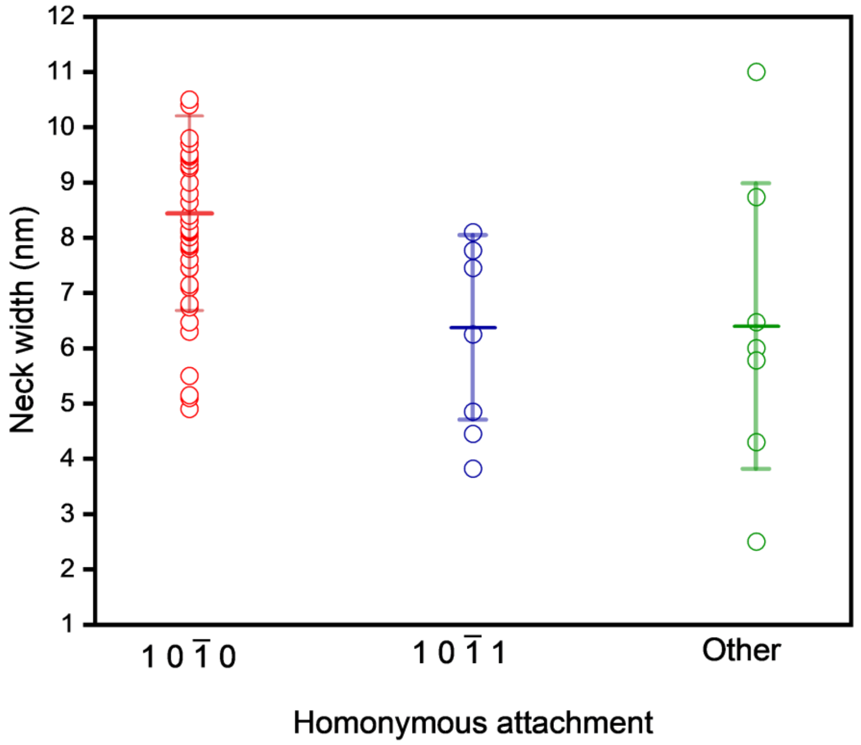

FIGURE S5: neck width histogram facet dependence. This figure presents the histogram of neck width as a function of attachment through different facets for CdSe/CdS core/shell quantum dots (CQDs) with a size of 2.2/5.8 nm, diameter is 16 nm. In cases of strong coupling, the neck is greater than the QD's radius.

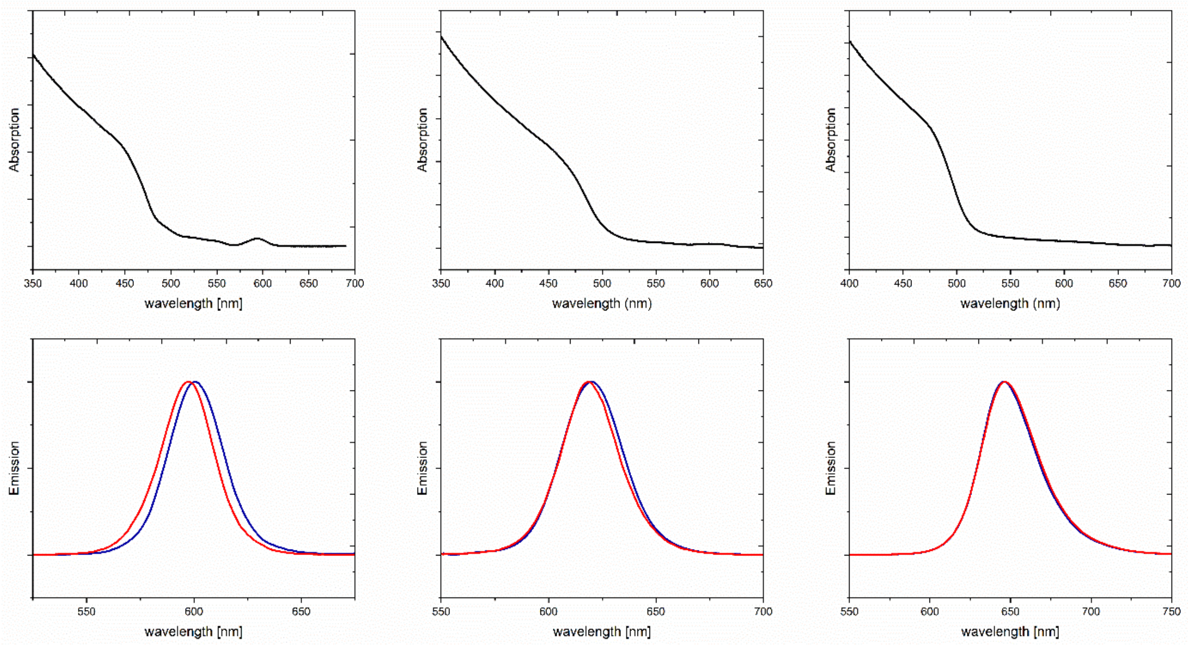

FIGURE S6: CQD molecules coupling effect. The coupling effect is manifested by the energy red shift of the CQD molecules compared to the individual CQDs. Panels (a-c) show the absorption spectra of CdSe/CdS core/shell QDs with sizes 1.4/2.1 nm, 1.2/4.3 nm, and 2.2/5.8 nm, respectively. The degree of coupling is evident in the red shift observed in the emission spectra (d-f), indicating changes between the CQDs (blue) *and the CQD molecules (red).*

	a_1	a_2	a_3	a_4
Cd	-39.5761	1.0205	-0.1361	1.6688
Se	5.7389	4.3792	1.2123	0.3197
S	5.4875	4.3685	1.2135	0.2915

TABLE S1: Psedopotential parameters for the fitting function. All parameters are in atomic unit.

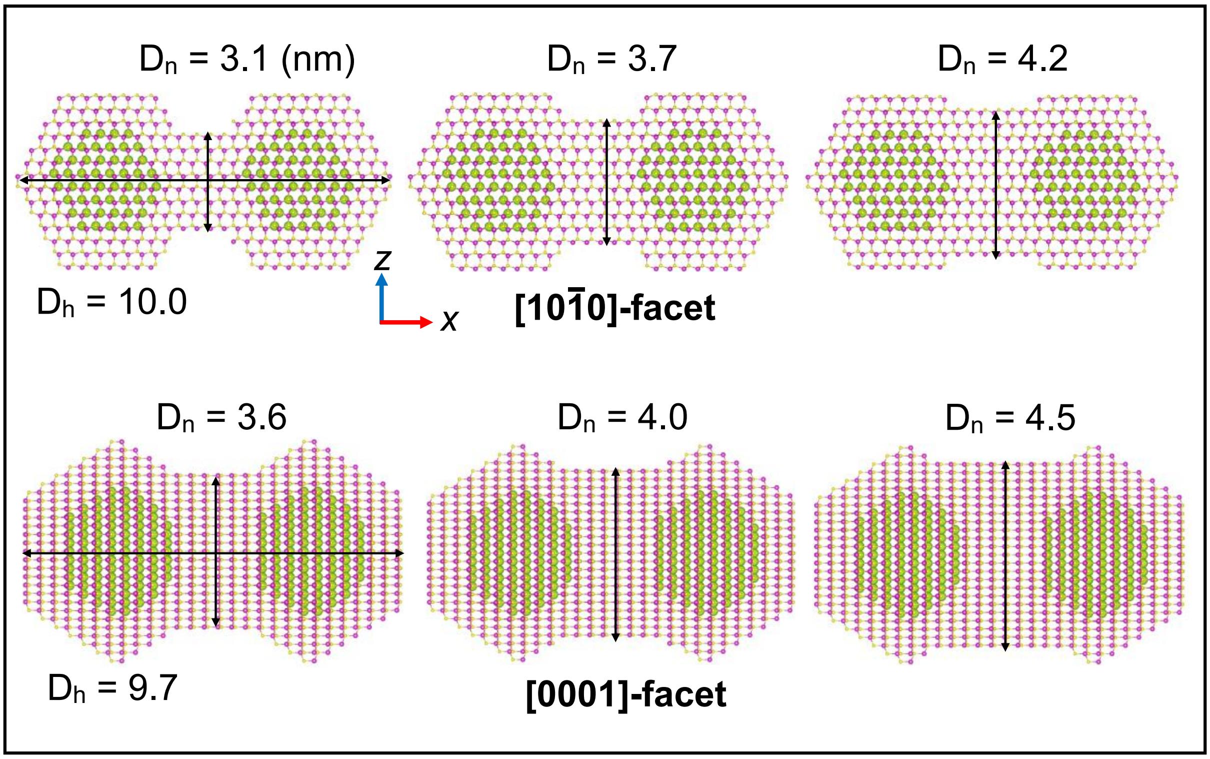

FIGURE S7: CQD molecule configurations for Figure 5a. The configurations for calculating energy redshifts in Figure 5a of the main text. The CQD molecules are constructed by attaching two monomers through either [10$\bar{1}$0] or [0001] facet. The neck width D_n is widened through adding CdS monolayers and the reported value is averaged in two directions.

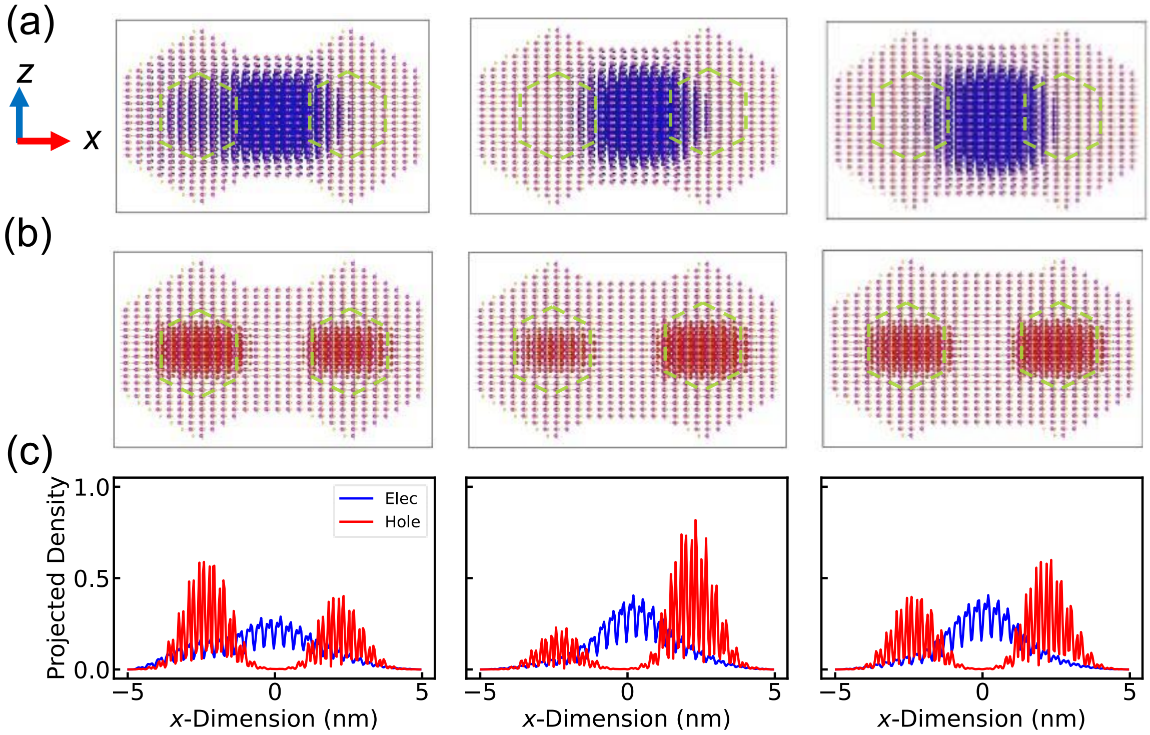

FIGURE S8: Interacting electron and hole densities for CQD molecules with different neck. The projected interacting electron and hole densities from the lowest excitonic density $|\psi_1(\mathrm{r}_e, \mathrm{r}_h)|^2$ of orientation [0001] with different neck sizes are shown in panel (a) and (b) respectively. The electron density is localized in CdS shell between the two monomers when the neck is wide. The hole density is localized in the CdSe core region (indicated by green circle). Panel (c) shows the projected interacting densities along the *x*-direction.

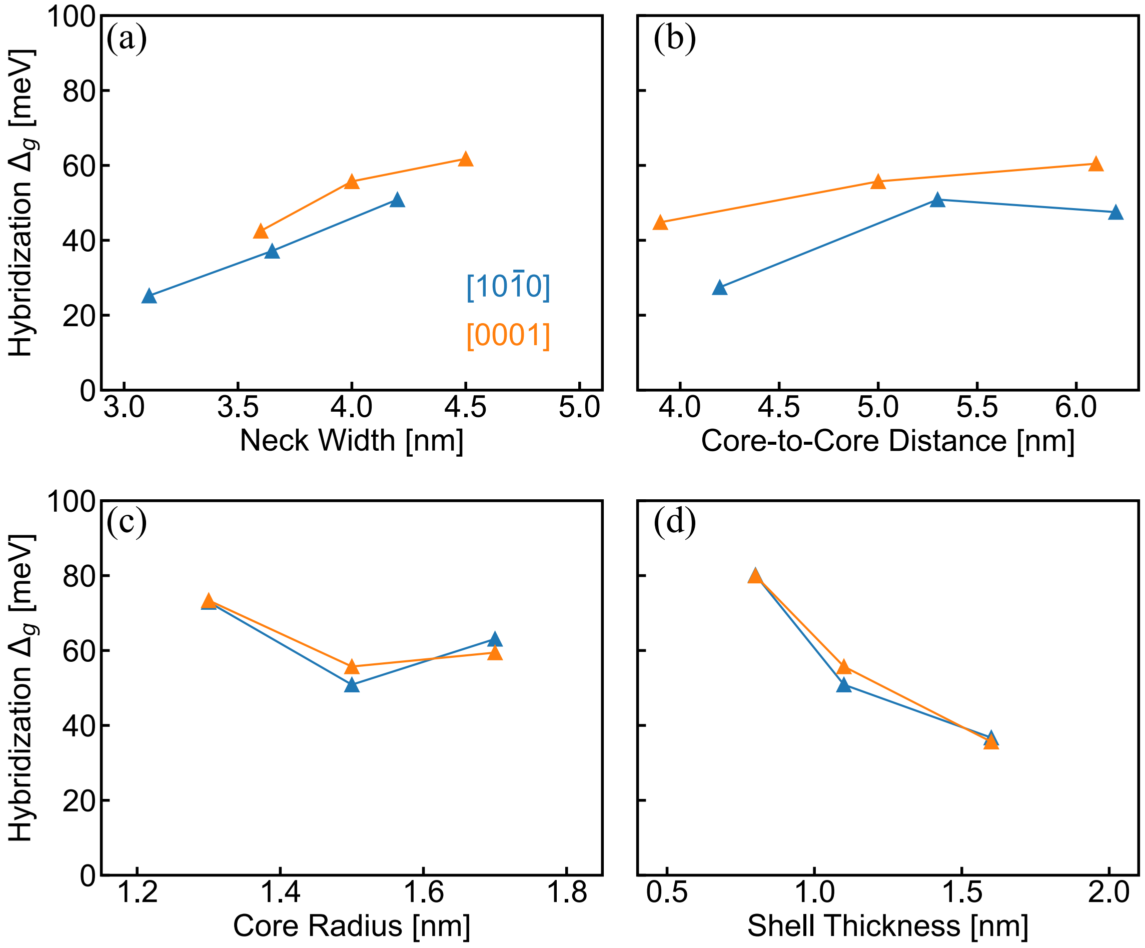

FIGURE S9: Contribution of non-interacting electronic couplings. The fundamental gap hybridization Δ_g for the systems shown in Figure 5 of the main text.

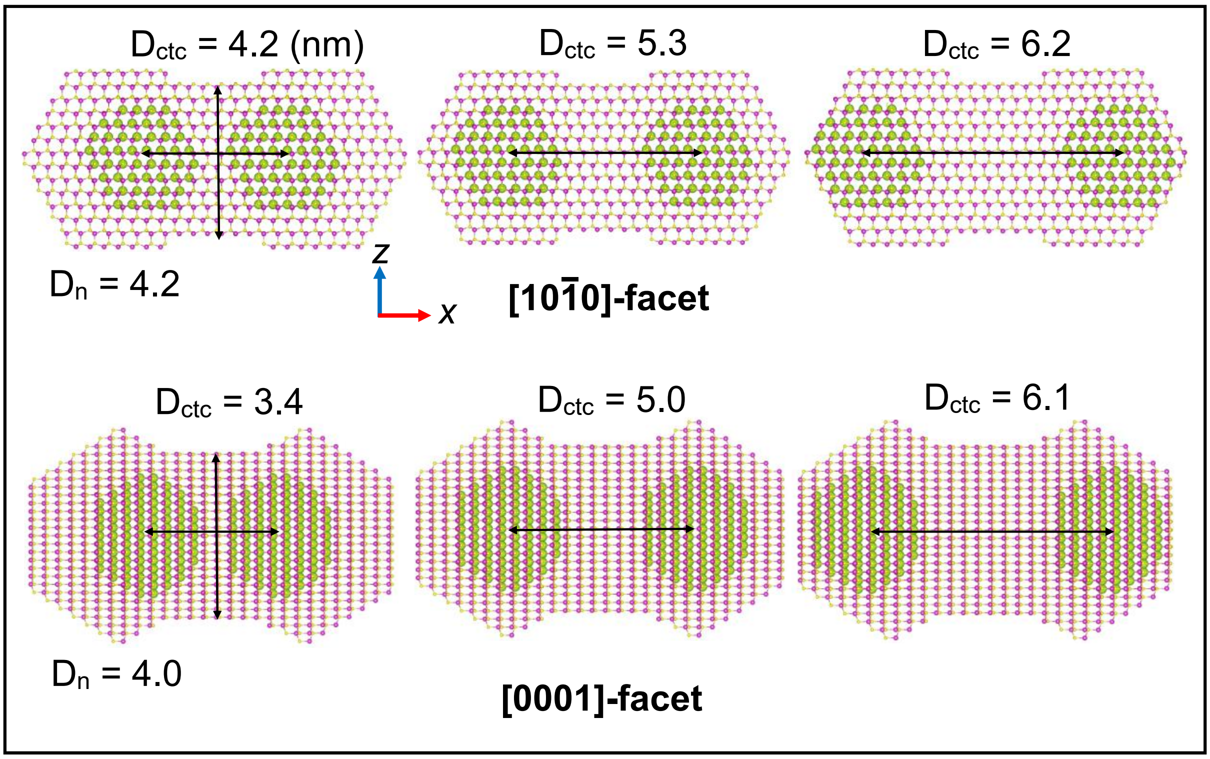

FIGURE S10: CQD molecule configurations for Figure 5b. The configurations for calculating energy redshifts in Figure 5b of the main text. The CQD molecules are constructed by attaching two monomers through either $[10\bar{1}0]$ or $[0001]$ facet with a fixed neck width D_n. The core-to-core distances D_{ctc} is varied through displacing the CdSe core symmetrically.

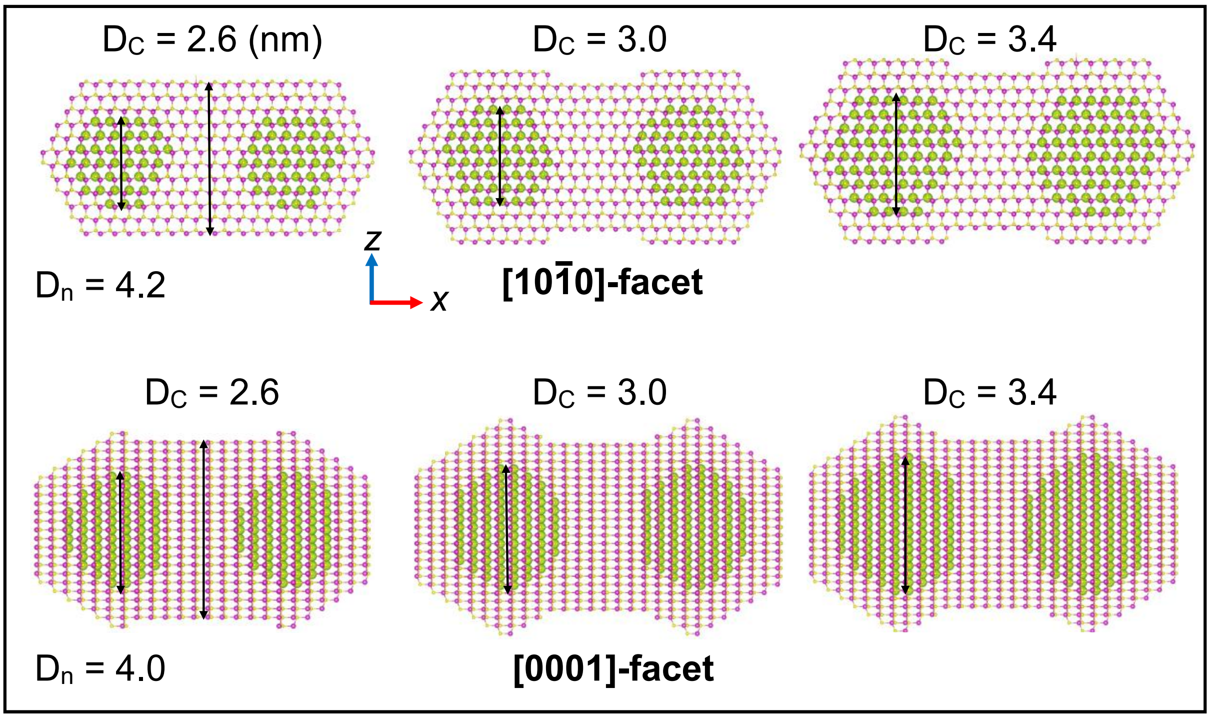

FIGURE S11: CQD molecule configurations for Figure 5c. The configurations for calculating energy redshifts in Figure 5c of the main text. The CQD molecules are constructed by attaching two monomers with CdSe core of diameter D_c through either $[10\bar{1}0]$ or $[0001]$ facet with a fixed neck dimension D_n.

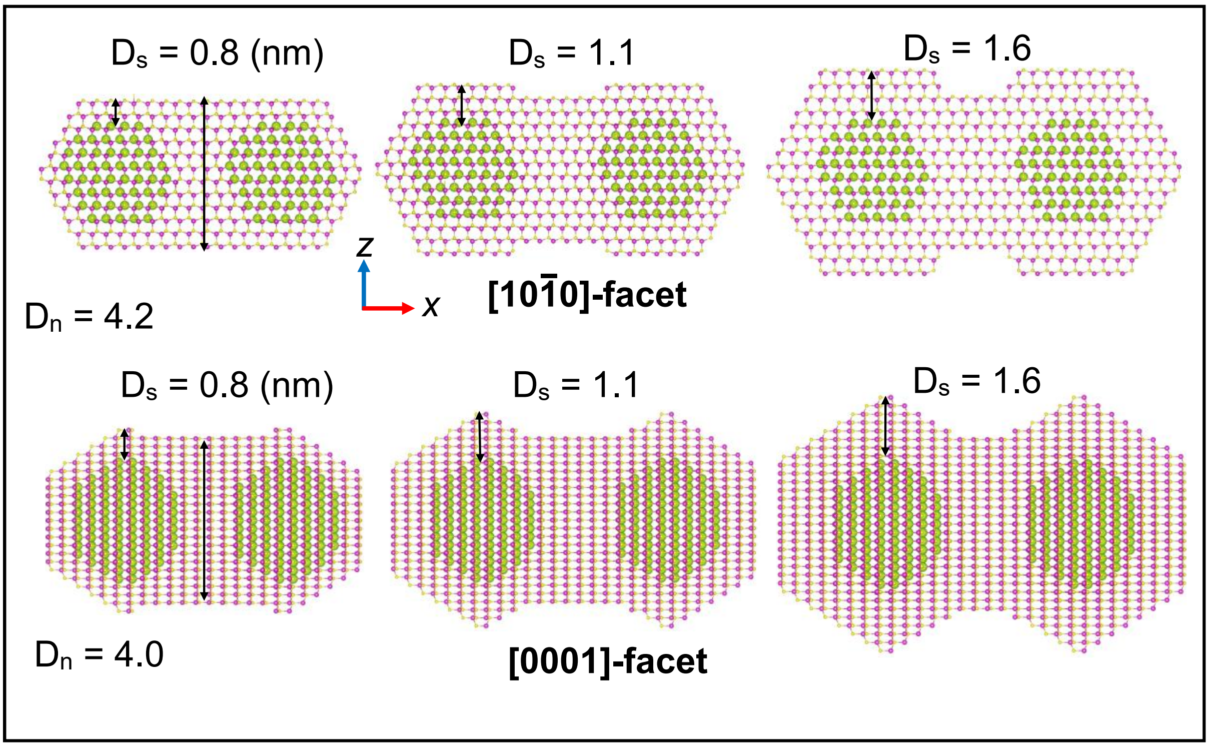

FIGURE S12: CQD molecule configurations for Figure 5d. The configurations for calculating energy redshifts in Figure 5d of the main text. The CQD molecules are constructed by attaching two monomers with CdS shell of thickness D_s through either [10$\bar{1}$0] or [0001] facet with a fixed neck dimension D_n.
